# Telomeres and Subtelomeres Dynamics in the Context of Early Chromosome Interactions During Meiosis and Their Implications in Plant Breeding

**DOI:** 10.3389/fpls.2021.672489

**Published:** 2021-06-04

**Authors:** Miguel Aguilar, Pilar Prieto

**Affiliations:** ^1^Área de Fisiología Vegetal, Universidad de Córdoba, Córdoba, Spain; ^2^Plant Breeding Department, Institute for Sustainable Agriculture, Agencia Estatal Consejo Superior de Investigaciones Científicas (CSIC), Córdoba, Spain

**Keywords:** crops, wheat, terminal chromosome regions, chromosome recognition, homologous pairing, recombination, meiosis

## Abstract

Genomic architecture facilitates chromosome recognition, pairing, and recombination. Telomeres and subtelomeres play an important role at the beginning of meiosis in specific chromosome recognition and pairing, which are critical processes that allow chromosome recombination between homologs (equivalent chromosomes in the same genome) in later stages. In plant polyploids, these terminal regions are even more important in terms of homologous chromosome recognition, due to the presence of homoeologs (equivalent chromosomes from related genomes). Although telomeres interaction seems to assist homologous pairing and consequently, the progression of meiosis, other chromosome regions, such as subtelomeres, need to be considered, because the DNA sequence of telomeres is not chromosome-specific. In addition, recombination operates at subtelomeres and, as it happens in rye and wheat, homologous recognition and pairing is more often correlated with recombining regions than with crossover-poor regions. In a plant breeding context, the knowledge of how homologous chromosomes initiate pairing at the beginning of meiosis can contribute to chromosome manipulation in hybrids or interspecific genetic crosses. Thus, recombination in interspecific chromosome associations could be promoted with the aim of transferring desirable agronomic traits from related genetic donor species into crops. In this review, we summarize the importance of telomeres and subtelomeres on chromatin dynamics during early meiosis stages and their implications in recombination in a plant breeding framework.

## From Genome to Chromosomes

A genome is the genetic information of a living organism. In eukaryotic organisms, like plants, the genetic information is carried by chromosomes, within the cell nucleus. A chromosome is made up by a supramolecular structure, called chromatin, which is a complex of a linear DNA molecule associated with several different proteins. Chromatin structure displays a multidimensional architecture. At its basic organizational level, a section of about 146 bp of the linear DNA molecule is wrapped around a canonical set of eight monomers (two copies of H2A, H2B, H3, and H4). The term nucleosome is used to describe the basic chromatin section (McGinty and Tan, 2015). Beyond the nucleosome scale, the chromatin fiber of around 10 nm diameter is further organized as an array of nucleosomes, with the participation of histone H1, a linker between adjacent nucleosomes, and packed in a higher order more compacted structure to form the so called 30 nm fiber that is in turn organized into folds of 150–200 kbp with an average diameter of 250 nm in interphase chromosomes, up to 850 nm in more compacted metaphase chromosomes (Dixon et al., 2016). Both the molecular composition and architecture of chromatin are not static but dynamic. Besides the intrinsic variable nature of the DNA sequence, chromatin molecular variations are the result of DNA methylation and demethylation, post-translational modifications of histones (including acetylation, methylation, phosphorylation, polyADP-ribosylation, and ubiquitination), replacing of canonical histone proteins by other non-canonical forms, and incorporation/elimination/modification of other non-histone proteins. This dynamical molecular composition of chromatin determines its organization and the state of compaction both at local and overall chromosome level, which is intimately related with its functionality around the whole cell cycle.

Microscopy observations of different intensity of chromatin staining allowed the distinction between darker and lighter stained regions of chromosomes, called heterochromatin and euchromatin, respectively (Heitz, 1928). Molecular analyses of chromatin revealed a correlation between the DNA sequence and the state of chromatin, being heterochromatin more densely packed, rich in repeat sequences and poor in genes, while euchromatin is all the way around, more loosely packed, poor in repeats and rich in genes.

Molecular differences of chromatin at the DNA level are also due to DNA modifications. Although adenine can also be modified by methylation, the most frequent DNA modification is cytosine methylation (5 mC). In plants, an RNA-directed DNA methylation machinery is responsible for *de novo* DNA methylation (Law and Jacobsen, 2010). DNA methylation status is the overall result of *de novo* methylation, maintenance of methylation, and active demethylation. Plants have a unique mechanism of DNA demethylation based on DNA glycosylases that excise and replace 5mC through a base excision repair pathway (Parrilla-Doblas et al., 2019). Regulation of transposon silencing, gene expression, and chromosome interactions is achieved by DNA methylation. This mechanism is particularly relevant in plant development, reproduction, and responses to biotic and abiotic stress conditions (Parrilla-Doblas et al., 2019).

The protein part of chromatin is also subject to important modifications. There are multiple isoforms of histones that can replace the canonical ones with a profound impact on chromatin functionality (Koyama and Kurumizaka, 2018). In plants, except for histone H4, all the core histones have several isoforms that eventually replace the canonical forms. These variant forms have properties that confer them different roles in DNA repair, gene switching, meiotic recombination, and chromosome segregation (Malik and Henikoff, 2003).

A thorough analysis of histone genes in the model plant *Arabidopsis thaliana* revealed a complex system of multiple gene families. While H4 is represented by a gene family that encodes an identical protein, the rest of histones gene families (H1, H2A, H2B, and H3) include genes that code for different isoforms. For histone H1, H2A, and H2B, each gene encodes a unique histone variant, while several genes encode H3.1 and H3.3 proteins. A total of 3 H1, 13 H2A, 11 H2B, 15 H3, and 8 H4 genes have been identified (Probst et al., 2020).

The main variants of H1, namely H1.1 and H1.2, are considered the canonical forms and their main function is related to chromatin compaction. H1.3, however, is necessary for adequate response to abiotic stress. Under non-stressed conditions, H1.3 expression is localized to a few cell types such as guard cells, but is strongly induced by abscisic acid, drought, and limited light (Rutowicz et al., 2015).

H2A.X is involved in DNA repair. H2A.W variants were initially believed to be exclusively involved with H3K9me2 and cytosine methylation, and with transposable element silencing. Today, an overall picture arises that plant H2A.W variants might play a role analogous to mammal KAP1 and HP1, with essential roles in cell differentiation and development (Lorković et al., 2017). H2A.Z variant has been related with metabolism (Yu et al., 2016) and with many physiological processes, including development (Jarillo and Piñeiro, 2015) or stress (Asensi-Fabado et al., 2017).

Several H2B variants are present in plants, as shown in *Arabidopsis*, where 11 different variants have been described (Probst et al., 2020). However, the genome distribution and possible functions remain unknown for most of them.

H3 histone is also present in multiple variants in plants. The *Arabidopsis* genome contains 15 genes coding for histone H3 representing H3.1, H3.3, CenH3, and other atypical variants. Five H3.1 genes are specifically expressed in S-phase and seem to be incorporated into nucleosomes during DNA synthesis (Okada et al., 2005; Jiang and Berger, 2017). There are three H3.3 genes and the protein deposition in nucleosomes seems to be independent of DNA-synthesis, since their expression is ubiquitous, even in non-proliferating cells (Okada et al., 2005). CenH3 is the third major H3 variant, coded by a single gene. It specifies the centromere and localizes to a specific subset of the centromeric 180 bp repeats (Nagaki et al., 2003).

Regarding their genome-wide distribution, H3.1 and H3.3 show important differences.

H3.1 is enriched in heterochromatic regions, while H3.3 is preferentially located at chromosome arms (Stroud et al., 2012). H3.3 accumulates in the 3' regions of transcribed genes and its distribution correlates well with transcriptional activity (Stroud et al., 2012), but it is also found in other regions, including telomeres (Vaquero-Sedas and Vega-Palas, 2013) and rDNA repeats (Duc et al., 2017).

In summary, the conformation of chromatin architecture at different levels seems to be greatly dependent on the substitution of core histones by replacement variants, and this, in turns, have profound functional implications that affect the whole plant life cycle.

Histones have unstructured N-terminal domains that protrude from the nucleosome core. Both nucleosome core and N-terminal domains can be post-translationally modified (Cosgrove and Wolberger, 2005). Besides methylation, post-translational modifications (PTMs) include acetylation, phosphorylation, and others. Methylation can have either permissive or repressive effects, while acetylation is related with chromatin activation. After the histone code theory, gene expression is affected by specific histone modifications (Strahl and Allis, 2000). More recent studies have revealed a complex and dynamic landscape of histone posttranslational modifications (PTMs) with multiple modifications added and removed from the same histone tail of the same nucleosome (Lee et al., 2010). The overall PTMs state of histones has a deep impact in chromatin architecture, and in turn in metabolism, cell differentiation, development, and response to environmental changes and stress conditions (Leung and Gaudin, 2020).

Besides the modifications caused by histone exchanging or by PTMs, chromatin can be remodeled by nucleosome mobilization to diverse DNA locations or removed by ATP-dependent remodeling enzymes. These remodeling factors are important for gene expression since they control the access of the transcription machinery through common mechanisms that include DNA unwrapping from the nucleosome core and DNA loop translocation along the nucleosomes (Saha et al., 2006; Zofall et al., 2006).

Chromatin is a dynamic multimolecular complex that shows a variable level of compaction and condensation/relaxation around the cell cycle. Many non-histone proteins interact with chromatin in a dynamical way so that they can support or remodel chromatin architecture conferring specific properties to the resulting structure. These changes affect local chromatin architecture, chromosome organization, and chromosome packaging, as well as DNA functionality, and it obviously has an influence on chromosome pairing and recombination.

Structural maintenance class of proteins (SMC) are a group of non-histone proteins, some of them having ATP-binding sites and enzymatic properties, that are essential for chromosome condensation, sister chromatid cohesion and segregation. Cohesins form a ring-shaped complex that support cohesion of sister chromatids, a fundamental mechanism for chromosome segregation. The cohesin complex is a ring composed of SMC1, SMC3 (two SMC subunits), and an α-kleisin, which recruits a fourth SMC subunit (SCC3). To achieve cohesion, the cohesin complex entraps DNA molecules. This process is regulated by other cohesin-binding proteins and modifying enzymes around the cell cycle (Nishiyama, 2019). Condensins form complexes that support chromatin compaction and packaging into chromosomes. They are a heterodimer of SMC2 and SMC4, two structural maintenance proteins that associate with specific regulatory subunits (Mainiero and Pawlowski, 2014). Both condensins and cohesins play important roles in chromosome organization to ensure genome stability.

Genetic insulator proteins like CTCF (CCCTC-Binding Factor), which play a relevant role in gene regulation by establishing topologically active domains (TADs) in animals, seem to be absent in plants. Neither TAD structures that function as insulated genomic units nor TAD border-binding insulator proteins have been reported. In maize and tomato, however, there are reports of long-range chromatin loops separating active and inactive domains, and other evidences of the existence of TADs or TADs-like domains in plants with large genomes (Liu et al., 2017; Wang et al., 2018). The study of TAD borders in plants suggests that TAD formation could be determined by the binding of specific TCP transcription factors and bZip proteins (Stam et al., 2019).

Another important class of non-histone proteins is constituted by the high mobility group (HMG) proteins. HMGA proteins have 4 A/T-hook DNA-binding motifs, are structurally flexible and bind A/T-rich DNA stretches. They could form higher-order transcription factor complexes through multiple protein-protein and protein-DNA interactions. HMGB proteins present a single HMG-box DNA-binding domain. They recognize specific DNA structures with no sequence specificity to enhance the structural flexibility of DNA and enable the assembly of nucleoprotein structures that control transcription and recombination (Grasser, 2003).

As a group, non-histone proteins have a relevant role in chromatin compaction to achieve higher-order chromatin structures as well as regulating its dynamical architecture, which has deep consequences on gene expression. At a global scale, these chromatin interactions regulate several processes including DNA repair, cell cycle, reproduction, differentiation, and multiple aspects of plant development.

## Nuclear Architecture in Interphase and Meiosis

The dynamic nature of chromatin and chromosomes is evidenced by the different changes that they experience around the cell cycle. These changes affect not only the molecular composition of chromosomes but also their local and global architecture, localization and arrangement within the nucleus, and their interactions with other nuclear and cellular structures. All these changes are relevant in the context of regulation of gene expression, cell differentiation, and development, response to environmental changes and stress conditions. And they are particularly relevant to understand the complex process of cell division, including mitosis and meiosis.

The studies on chromatin and chromosome dynamics, especially in meiosis, have been paid much more attention in the context of plant reproductive processes partly because the initial studies were based on visual observations through the light microscope, and the highly condensed chromatin during cell division is easier to visualize than in the interphase. The importance of plant breeding has also contributed to focus the study of chromosome dynamics during meiosis. However, during the last decades, newer and more powerful techniques have been developed such as fluorescence *in situ* hybridization (FISH), immunofluorescence-FISH, 3D FISH (Chaumeil et al., 2013), 3C, 4C, 5C, and Hi-C (high-throughput chromosome conformation capture; Dekker, 2006; Shaw, 2010; De Wit and de Laat, 2012; Dekker et al., 2013), fluorescence recovery after photobleaching (FRAP; White and Stelzer, 1999; Phair and Misteli, 2001), Covalent Attachment of Tagged Histones to Capture and Identify Turnover (CATCH-IT; Deal et al., 2010), or Single-Particle Tracking (SPT; Straight et al., 1996; Belmont et al., 1999). With the aid of these techniques, the attention has been extended to the study of chromosomes around the cell cycle, particularly during the interphase, having in mind the idea that the knowledge of chromosome dynamics during the interphase will also help to understand chromosome dynamics during mitosis and meiosis.

Multiple studies conducted in the model plant *A. thaliana* and other species have allowed the elucidation of interphase chromatin organization. Chromatin is relatively relaxed and decondensed in interphase nuclei. However, its arrangement within the nucleus is far from being random. Each chromosome seems to occupy a specific region within the nucleus. This idea was initially suggested by Rabl (1885). Boveri (1909) introduced the concept of chromosome territory (CT). CTs were experimentally confirmed by FISH using chromosome-specific probes in human cells (Manuelidis and Borden, 1988). Chromosome territories were also demonstrated in the model plant *A. thaliana* by Lysak et al. (2001).

In *Arabidopsis*, chromosomes are organized in a way that all telomeres are clustered around the nucleolus and tend to associate with it, as an anticipation of homologous pairing in meiosis. Centromeres, however, are dispersed toward the periphery of the nucleus (Armstrong et al., 2001; Fransz et al., 2002). In this species, whose genome is very small (135 Mbp), heterochromatin around the centromeres shows dense bodies called chromocenters. These chromocenters are inactive chromatin regions, enriched in sequence repeats, from which euchromatic regions arise as loops that give a characteristic rosette structure to *Arabidopsis* chromosome territories (Fransz et al., 2002). The positioning of *Arabidopsis* chromosomes relative to each other seems to be random (De Nooijer et al., 2009), except for those chromosomes that carry nucleolar organizing regions (NORs), which contain multiple copies of rRNAs arranged in tandem (Pecinka et al., 2004). The association of NORs with the nucleolus must cause the clustering of all the chromosomes that contain NORs. Specific interchromosomal interactions and dynamics can be influenced by this configuration in *A. thaliana*. A recent study in autotetraploid *Arabidopsis* suggests that chromosome territories are somehow independent (Zhang et al., 2019).

In other plant species, some of them with large genomes such as wheat (14.5 Gbp), chromosomes display the so called Rabl configuration, where the chromosome is folded at the centromere so that both telomeres and subtelomeric regions are close together. Telomeres are grouped at one pole and centromeres are grouped at the opposite pole of the nucleus (Anamthawat-Jonsson and Heslop-Harrison, 1990; Cowan et al., 2001; Doğan and Liu, 2018). Beyond the existence of CTs, the study of polyploid organisms like wheat and *Brassica napus* has shown that chromosomes of the different subgenomes are not intermingled but segregated, so that all chromosomes of a subgenome occupy a kind of genome territory, being the interactions among chromosomes of the same subgenome more probable and intense. In the case of bread wheat, its genome includes three subgenomes (A, B, and D) that would occupy three different genome territories within the nucleus (Concia et al., 2020).

Interphase chromosomes are not just occupying a chromosome territory; they interact with other macromolecules and structures. The interactions of telomeres and centromeres with lamina nuclear envelope and nucleolus proteins allowed the definition of two broad chromosome domains that have profound functional implications. Lamina-associated domains (LADs) are extensive chromatin stretches that interact with a network of lamin fibers near the inner nuclear membrane (Guelen et al., 2008). Nucleolus-associated chromatin domains (NADs) are chromatin regions in contact with the nucleolus (van Koningsbruggen et al., 2010). Several studies during the last 20 years have allowed the identification of multiple factors that seem to be involved in the positional control of chromosomes, through their interaction with centromeres and telomeres during the interphase in plants. Besides the structural maintenance of chromosome (SMC) complexes, these factors include a few proteins of the nuclear membrane and the nucleolus (Oko et al., 2020) that have allowed the definition of LADs and NADs also in plants.

Plant LADs involve both telomeric and centromeric domains. Telomeres are localized at the nuclear periphery during the interphase (Dong and Jiang, 1998), with some exceptions, such as *Arabidopsis*, where the nucleolus interacts with telomeres (Roberts et al., 2009). As it is the case in yeasts, there must be a few membrane proteins playing an important function in the positioning of telomeres at the nuclear periphery, though none have been identified in plants yet (Ebrahimi and Cooper, 2016). In maize, ZmSUN2 seems to be involved in the localization of telomeres at the periphery of the nucleus in meiosis, but the function of ZmSUN2 during the interphase remains unknown (Murphy et al., 2014). In plants, as in other eukaryotes, centromeres are anchored at the periphery of the nucleus (Muller et al., 2019). Regardless of Rabl or non-Rabl configuration of plant chromosomes, the anchoring of centromeres at the periphery of the nucleus could fix their position in the interphase. The knowledge of the protein factors involved in centromere anchoring to the nuclear periphery is more extensive. In *Arabidopsis*, whose chromosomes show a non-Rabl configuration, SUN proteins seem to maintain the centromere position near the nuclear periphery (Poulet et al., 2017) and might function as a linker of Nucleoskeleton and Cytoskeleton complex (LINC). This complex, formed by proteins located at the inner and outer nuclear membranes, links the lamina with the cytoskeleton (Fernández-Álvarez et al., 2016). Also, in *Arabidopsis*, CROWDED NUCLEI 1, a putative SUN-interacting protein (Graumann, 2014), mediates the tethering of chromosome arms and centromeric heterochomatin at the periphery of the nucleus (Hu et al., 2019). Considering that *Schizosaccharomyces pombe* Lem2 protein is involved in the positioning of interphasic telomeres and centromeres (Barrales et al., 2016; Fernández-Álvarez et al., 2016), the possibility exists that the positioning of plant centromeres and telomeres could also be controlled by CRWNs, condensin II and other proteins implicated in centromere positioning. As proposed by Oko et al. (2020), the fact that Lem2 prevents the loss of Rabl-type configuration in *S. pombe* during interphase suggests the existence of a mechanism that keeps centromere clustering in plants with Rabl configuration with telomeres at the opposite side (Hou et al., 2012; Barrales et al., 2016; Fernández-Álvarez et al., 2016).

Regarding plant NADs, it is known that *Arabidopsis* chromosomes are organized in a way that all telomeres are clustered around the nucleolus and tend to interact with it. NADs were identified in isolated nucleoli (Pontvianne et al., 2016). NADs are rich in transposable elements and poorly expressed genes, what agrees with the fact that interactions with the nucleolus occur through telomeric and subtelomeric regions. *Arabidopsis* NUCLEOLIN 1 (NUC1) is involved in telomere-nucleolus associations and seems to be essential to keep telomere length (Pontvianne et al., 2016). When compared to animals, our knowledge of plant LADs and NADs is still very limited. We do not know their precise limits, all the numerous factors involved in their organization and control, and how they evolve around the cell-cycle, and in the context of development and changing environmental conditions (Pontvianne and Liu, 2020).

In interphase nuclei, chromosomes not only interact with other macromolecules and nuclear structures within the nucleus, but they also interact among them either directly or through proteins or more complex structures. Besides its basic function as information storage, the whole genome should also be considered as a physical structure with internal forces being exerted and transmitted within chromosomes and among chromosomes, and also from and to the rest of the nucleus and the whole cell. The limited volume of the nucleus enables this interaction, which is already supported by the non-random distribution of chromatin, with a precise distribution of heterochromatic and euchromatic regions, TADs, LADs, NADS, and CTs. Transcription factories, trans-regulation of gene expression, replication machineries, and DNA repair mechanisms reveal the existence of interchromosomal interactions during the interphase and explain the connection between organization and function of the whole genome.

In a species with a small genome like *A. thaliana*, telomeres are clustered at the nucleolus during interphase (Armstrong et al., 2001). The interaction between NORs and nucleolus determines the nonrandom association of chromosomes and might have direct effects on interchromosomal interactions and dynamics. Associations were found to be basically random among chromosomes 1, 3, and 5, while the associations of NOR-bearing chromosomes 2 and 4 were more frequent. The association of NOR-bearing chromosomes would be due to the interaction of both homologs with a single nucleolus, as found in expanded leaves and root meristems (Cremer et al., 2001; Pecinka et al., 2004; Berr and Schubert, 2007). A similar picture of chromosome associations was found in *Arabidopsis lyrata*, another *Brassicaceae* with larger genome (Berr et al., 2006).

As mentioned before, a Hi-C study of autotetraploid *Arabidopsis* showed increased interchromosomal interactions and reduced intra-arm interactions in comparison with a diploid strain. These increased interchromosomal interactions were localized around centromeres, while decreased intertelomeric interactions were observed among all chromosomes in autotetraploid plants. The results of this study suggested that autotetraploid *Arabidopsis* had less compacted chromosome arms and that interchromosomal interactions presented higher strengths in the autotetraploid compared to the diploid strain. The increased interchromosomal interactions were unspecific and no preferential interactions were found between any given pair of heterologous chromosomes (Zhang et al., 2019).

A similar Hi-C study in rice (*Oryza sativa* L.) revealed that chromosomes occupy specific territories, and they interact preferentially with certain chromosomes (Dong et al., 2018). Two sets of chromosomes (1 through 5 and 10 through 12) interacted preferentially within the set, while the remaining chromosomes (6, 7, 8, and 9) did not show apparent associations at all. These observations suggest that chromosomes that show more frequent associations must be physically closer in space within the nucleus. The difference between rice and *Arabidopsis* could be explained because rice has a larger and more complex genome with a larger number of chromosomes (Dong et al., 2018).

The observed interactions among chromosomes could be at least partly explained because of the spontaneous Brownian motion within the constrained space of the nucleus. However, we must also consider the importance of other more intense forces that are delivered during the multiple active processes that concern chromatin and chromosomes at work. One of these processes is transcription. Chromatin conformation determines the access of the transcription machinery to the DNA (Kouzine et al., 2014). The arrangement of CTs that allows the approach of certain chromosome regions led to the idea of transcriptional factories, hundreds of sites with an especially high transcriptional activity (Sexton et al., 2007; Sutherland and Bickmore, 2009).

The distribution of transcriptional activity across chromosomes underlies many aspects of large-scale nuclear architecture including interchromosomal associations (Agrawal et al., 2020; Menon, 2020). *Arabidopsis* transcriptionally active genes do not associate in transcription factories (Liu et al., 2016). The picture is different in species with more complex genomes. In plants with large genomes like hexaploid wheat, transcription factories have been shown to allow both intra‐ and interchromosomal contacts associated to RNA polymerase involving multiple genes displaying similar expression levels (Concia et al., 2020).

Replication is another chromosomal process that seems to imply interchromosomal interactions. In plants, there is little information about chromosome interactions during replication. In *Arabidopsis*, however, they found a correlation between genomic regions that replicate during the interphase and genomic features, chromatin state, accessibility, and chromosomal interactions. They suggest that sequences that are close together tend to replicate at the same time (Concia et al., 2018). A similar picture was found in a larger study involving time and position parameters of DNA replication in several *Poaceae* including wheat, oat, rye, barley, *Brachypodium*, rice, and maize (Němečková et al., 2020).

DNA repair also implies both intra‐ and interchromosomal interactions. DNA repair is extremely important in plants, due to their sessile nature and their exposure to multiple mutagenic agents like ionizing radiation, heavy metals, and other types of biotic and biotic sources of stress, besides the endogenous processes that can result in DNA damage. Among all the types of damages that DNA can suffer, double strand break (DSB) is the most severe. The major mechanism of DSB repair in somatic plant cells is non-homologous end joining (NHEJ), but some DSBs are fixed by homologous recombination (HR). NHEJ may result in loss or change of information due to deletions, inversions, translocations, or insertion of sequences from elsewhere in the genome (Lieberman-Lazarovich and Levy, 2011; Knoll et al., 2014).

DNA repair by HR can follow two different mechanisms: single-strand annealing (SSA) and synthesis dependent strand annealing (SDSA). SDSA is the major mechanism of conservative HR repair in plant somatic cells (Knoll et al., 2014). Sister chromatids can be used as template in the S and G2 phases, and this is the most efficient kind of template. However, DNA repair by SDSA HR is also possible in interphase somatic cells between homologous chromosomes, or also between homoeologs (in the case of polyploid plants), and even between heterologous chromosomes, though it occurs at very low frequencies. In tomato, induced allele dependent DSB repair has been proposed (Gisler et al., 2002; Knoll et al., 2014).

In humans, it is known that during G_0_-phase, as early as 5 min after DNA damage induced by ionizing radiation, homologous chromosomes interact at the DSB sites, what could be explained by the existence of a fast mechanism to localize homologous chromosomes where DSBs are generated (Gandhi et al., 2013). In plants, the precise mechanisms of chromosome approach and interaction to allow DSB repair by HR are not known. Recombination between homologs in somatic tissues is not well-documented because of intrinsic difficulties. In tobacco, it was shown that somatic HR is not frequent in the absence of DSB induction (Carlson, 2016). More recently, Filler-Hayut et al. (2017) showed that the induction of DSBs in tomato somatic cells *via* CRISPR-Cas9 increases the frequency of homologous contact and recombination between homologous chromosomes, demonstrating that the meiotic HR machinery is not necessary for DSB-induced homologous recombination. Even though there is not enough information, concerted chromatin modifications seem to determine DSBs repair through the repair machinery and repair factors. Chromatin changes are also correlated with the movement of repair sites to the periphery of the nucleus for HR repair of DSBs in heterochromatic DNA (Kim, 2019).

In plants, somatic homologous pairing and recombination is not frequent, though it is relevant in certain situations. The fact that multiple processes like transcription, genetic regulation, replication, and DNA repair allow interchromosomal contacts throughout the whole cell-cycle points to the relevance of all these processes to explain homologous chromosome pairing and recombination in the interphase, in somatic cells, as well as in reproductive cells and meiosis.

Transcription seems to be particularly relevant, since it could somehow set off somatic pairing of homologous chromosomes (Hiraoka, 2020). Homologous chromosomes, with identical chromosome architecture, display almost identical patterns of transcription factories and heterochromatin (Cook, 1997). Homologous chromosomes are thought to be joined at the transcriptional factories (Cook, 1997; Ding et al., 2010). Non-coding RNAs accumulate on their gene loci, and they could contribute to the association of homologous chromosomes through allelic loci (Ding et al., 2012).

In plants, though homologous chromosome pairing seems to be usually random and transient in somatic cells (Schubert and Shaw, 2011), there is evidence of constitutive homologous chromosome pairing at least in certain cell types. A study on the arrangement *Brachypodium distachyon* chromosomes in root cells interphase nuclei showed that the association of homologous chromosome arm CTs is more frequent than expected in a random arrangement of all chromosomes within the nucleus (Robaszkiewicz et al., 2016).

Although preceded by heterologous association through centromeres, homologous pairing along the entire chromosomes was also described in hexaploid wheat floral tissue prior to meiosis (Martínez-Pérez et al., 1999). The Rabl configuration of wheat chromosomes was observed in premeiotic cells (Naranjo, 2015a). This configuration is important for homologous recognition, since it facilitates homologs search and alignment (Pernickova et al., 2019). In rice, they found initial chromosome association between homologs in undifferentiated anther cells and xylem vessel cells. In wheat and related polyploids, however, initial association in undifferentiated anthers was found between non-homologous or related chromosomes, but not between homologs (Prieto et al., 2004b). All these observations support the concept that somatic homologous pairing might precede and contribute at least partially to meiotic homologous pairing and recombination, as already suggested (Gerton and Hawley, 2005).

## The Structure and Function of Chromosome Ends

Despite the evident relevance of multiple types of interchromosomal interactions occurring around the whole cell-cycle, chromosome ends (telomeres and subtelomeric regions) are known to be involved in specific homologous chromosome recognition and pairing during early meiosis, which are critical processes that allow chromosome recombination between homologs in later stages. In plant polyploid species, like wheat and maize, these terminal regions are even more important in terms of homologous chromosome recognition, due to the presence of homoeologs (equivalent chromosomes from related genomes) and the necessity to prevent pairing and recombination between non-homologous or homoeologous chromosomes.

The chromosomal reorganization that takes place at the beginning of meiosis seems to be rather ubiquitous in the process of homology searching in higher eukaryotes (Blokhina et al., 2019). Telomeric sequences are highly conserved. Chromosome ends cluster near the nuclear membrane to form a “bouquet” structure. This process facilitates homologs preliminary interactions and further pairing and recombination (Naranjo, 2014). Although telomeres interaction seems to assist homologous pairing and consequently, the progression of meiosis, other chromosome regions such as subtelomeres need to be considered, because telomere sequences are not specific for any chromosome. In addition, recombination operates at subtelomeres and, in some crop species, there seems to be a correlation between recombination and homologous pairing. A complete characterization including the DNA sequence, as well as chromatin composition, modifications, and 3D architecture, together with the molecules (proteins, RNA, etc.) interacting with it, is needed to get insight into the function of telomeres and subtelomeres in pairing and recombination.

In most eucaryotes, the telomere sequence is typically constituted by short G/C-rich repeats organized in tandem, with the G-rich strand 3' end toward the chromosome end, which often has a G-overhang that forms a single strand protrusion (Table 1). Some plant species like *A. thaliana* and other angiosperms do not have this overhang or at least not in all chromosomes (Kazda et al., 2012). The model plant *A. thaliana* was the first multicellular organism whose telomere sequence was cloned and characterized (Richards and Ausubel, 1988). In the majority of plant species, telomeres sequences are formed by arrays of tandem repeats of variable length from less than 1 kbp to tens of kbp, depending upon species, variety, organism, and chromosome (Fajkus et al., 1995a,b). The TTTAGGG telomeric repeat first found in *Arabidopsis* was considered ubiquitous among most plant species (Kilian et al., 1995), with a few exceptions as the unicellular green alga *Chlamydomonas reinhardtii*, whose telomeric repeat is TTTTAGGG (Petracek et al., 1990), and a group of Asparagales, where it is TTAGGG (Sykorova et al., 2003b; Weiss-Schneeweiss et al., 2004). But more recently, a higher variability around the formula (T_x_A_y_G_z_)_n_ has been found in many species of *Solanaceae*, *Amaryllidaceae*, *Lentibulariaceae*, and many algae (Peska and Garcia, 2020). This variability seems to be the result of evolutionary divergence. In monocots, telomeric sequences evolved from (TTTAGGG)*n* and a change to (TTAGGG)*n* occurred in *Iridaceae*, while (CTCGGTTATGGG)*n* sequence is found in *Allium* (Fajkus et al., 2019).

**Table 1:** General features of chromosome ends.

G-overhang	Telomere	Telomere-subtelomere junction	Subtelomere
Length = A few nucleotides	Length = Up to hundreds of bp	Length = Up to a few kbp	Length = Up to hundreds of kbp
3'-G_n_ Common, but absent in some chromosomes and species.	(TTTAGGG)*n* Common, but absent in some chromosomes and species.	Variable number of telomere degenerate repeats close to telomere repeats.	Highly polymorphic set of transposons, retrotransposons, low complexity DNA, genes including tRNAs, transcription factors, and metabolic genes with functions that are required for adaptation to the environment.
	(TTAGGG)*n* in Iridaceae (TTTTAGGG)n in *Chlamydomonas reinhardtii.* (CTCGGTTATGGG)*n* in *Allium.* (TxAyGz)*n* in Solanaceae, Amaryllidaceae, Lentibulariaceae, and many algae.	Tandem arrays of rRNA genes in some chromosomes of many species. BAAAA (B = C, T, G) and a poly-G stretch of about 32 bp in *Arabidopsis.*	Highly variable pattern of multiple sequence features (rice). Sets of specific repeats (barley and wheat). Large blocks of heterochomatin (rye).

The distal part of the telomere is changing continuously as a result of two opposing processes: a shortening due to the end-replication problem and exonucleolytic resection vs. an active elongation by the action of telomerase (Fitzgerald et al., 1999; Riha et al., 2001; Riha and Shippen, 2003). G-rich overhangs, besides other important functions, could act by invading the telomere contiguous duplex region so that a t-loop is formed. This would be a maintenance mechanism for the telomere protective cap (Cesare et al., 2003). Plant telomeres are maintained by telomerase, as it occurs in mammals (McClintock, 1939). Telomerase is expressed and active in meristematic cells of all growing tissues and organs such as apex, root tips, young leaves, inflorescences, flowers, and seedlings, while it is absent in completely differentiated and mature tissues (McClintock, 1941; Kilian et al., 1998). In most organisms, including plants, telomerase is formed by two elements: telomerase reverse transcriptase (TERT) and telomerase RNA (TR; Veldman et al., 2004; Hockemeyer et al., 2006). Telomerase also includes other proteins that are necessary for its function (Tommerup et al., 1994; Wu et al., 2006). TERTs are highly conserved (Sykorova and Fajkus, 2009), while TRs are very diverse both in sequence and length among most organisms. In plants, however, the TR gene is highly conserved (Fajkus et al., 2019). In plants, an HR-based telomerase maintenance mechanism (ALT) is probably active during early plant development (Bryan et al., 1997; Ruckova et al., 2008). ALT is dependent of t-loops, which resembles the first steps of HR (Griffith et al., 1999).

Chromosome ends are protected by proteins associated with telomeres. This protection implies the distinction between natural chromosome ends from accidental DNA breaks, avoiding the unwanted action of the repairing machinery on telomeres. In humans, this set of telomere-binding proteins, a complex known as shelterin, include TRF1, TRF2, POT1, and other proteins that interact with telomeres indirectly (De Lange, 2005, 2018). Besides its protective function, the shelterin complex also contributes to the recruitment of telomerase, the movement of the replication fork or the creation of t-loops (Procházková-Schrumpfová et al., 2019). Plant telomeres also have a shelterin-like complex, though its characterization is still incomplete. As putative components of this shelterin-like complex, TRB proteins were identified in *A. thaliana* (Schrumpfova et al., 2014) and maize (Marian et al., 2003). These proteins bind telomere DNA specifically both *in vitro* and *in vivo* and interact with the TERT subunit of telomerase (Peska et al., 2011; Schrumpfova et al., 2014; Zhou et al., 2018).

Plant Ku and POT1b proteins associate with TER2, a TR that is not necessary for telomere maintenance (Cifuentes-Rojas et al., 2011). Ku70/80 heterodimer plays an important role in the protection of blunt-end telomeres. Plants conserve all orthologs of scaffold box H/ACA of small nucleolar RNAs. A CST protein complex seems to be relevant for an efficient replication of plant telomeres. RTEL also takes part in the maintenance of the homeostasis of telomeres (Procházková-Schrumpfová et al., 2019).

We should also have in mind that most of the telomere chromatin is tightly packed in nucleosomes, being the nucleosomal spacing in telomeres shorter than elsewhere in the chromatin (Fajkus et al., 1995a,b). In addition, other telomere heterochromatin features are possible, since nearby regions form large heterochromatin blocks in many plants (Louis and Vershinin, 2005). This could be particularly relevant when the long rack of regular telomere repeats is absent. Some *Alliaceae* and *Solanaceae* have lost G-rich telomeric repeats (Sykorova et al., 2003a, 2006). In *Allium cepa* (onion), chromosome ends were proposed to contain satellite repeats and transposons (Pich and Schubert, 1998). Thus, maintenance of telomere structure in *Alliaceae* could imply an epigenetic mechanism as in *Drosophila*. In *Arabidopsis*, they reported telomerase-deficient mutants that lacked telomeric DNA but partially retained the ability to end-cap their chromosomes, suggesting the existence of an adaptive mechanism to the loss of telomeric DNA based on the formation of terminal heterochromatin blocks (Watson et al., 2005).

Telomeres are also subject to epigenetic modifications. Methyl-Cytosines (mCs) were detected in telomeric repeats of *Arabidopsis* (Ogrocka et al., 2014), *Nicotiana tabacum* (Majerova et al., 2011), and other plants (Majerova et al., 2014). Telomere homeostasis was altered because of a reduction of genomic DNA methylation in *Arabidopsis* (Xie and Shippen, 2018) but not in tobacco (Majerova et al., 2011), which reveals significant differences in the regulation of telomere homeostasis by methylation. The plant telomeric chromatin displays a dual epigenetic character, since chromatin was associated with heterochromatin and euchromatic marks in telomeres (Majerova et al., 2014; Sovakova et al., 2018). A general conclusion on the epigenetic state of telomeric chromatin is not possible since it is very dynamic and variable with the physiology of the organism. Epigenetic modifications are responsible for the regulation of telomere functioning (Procházková-Schrumpfová et al., 2019; Achrem et al., 2020).

In all organisms with a complex genome, including *Homo sapiens*, *Arabidopsis*, and wheat, the region that is closed to the telomere, is usually characterized by the presence of a variable number of telomere degenerate repeats (Aguilar and Prieto, 2020). Though a clear functional or structural definition is not available, the subtelomere is usually considered as the region extending from the telomere up to the first chromosome-specific sequence (Louis, 2014). A common feature supposed to be shared by plant chromosome ends is a region of highly repetitive and reorganized DNA before the first active gene (Alkhimova et al., 2004; Aguilar and Prieto, 2020). *Arabidopsis* chromosomes, however, have short and simple subtelomeric regions. Except for two chromosome ends, where telomeric tandem repeats are right adjacent to tandem arrays of rRNA genes, subtelomeres contain a few repeats of the sequence BAAAA (B = C, T, G) and a poly-G stretch of about 32 bp (Kuo et al., 2006). A recent study in wheat chromosome ends, however, did not show any characteristic pattern among five different chromosomes whose telomere-subtelomere border regions were studied. The characteristic features found in *Arabidopsis* were absent in wheat, but many different elements (genes, retrotransposons, transposons, tandem repeats, and low complexity DNA) were found, as in other plants (Table 1; Aguilar and Prieto, 2020).

Subtelomeres are highly polymorphic and, as a matter of fact, they are less conserved than chromosome ends. Genes are very abundant, and recombination is more frequent in these regions (Emden et al., 2019). These characteristics make it more difficult to analyze the actual role of subtelomeres in genome stability, replication, and also in chromosome pairing and recombination (Emden et al., 2019). Chromosome subtelomeric distal regions seem to play important roles in other processes including transcription, chromosome dynamics during meiosis, and the regulation of the cell cycle cell (Blackburn, 2005).

In most species, the analysis of subtelomeres has been focused on a distal segment of around 500 kbp (Mizuno et al., 2008). These studies revealed many differences among species. In *A. thaliana*, probably due to its small genome, subtelomeres seem to be short and rather simple, and their sequences are very variable among non-homologous chromosomes (Kuo et al., 2006). Rice subtelomeres showed a highly variable pattern of multiple sequence features (Mizuno et al., 2014), while rye subtelomeres contain large blocks of heterochomatin (Evtushenko et al., 2016). Sets of specific repeats were found in subtelomeres of some species including barley and wheat (Prieto et al., 2004a; Salina et al., 2009). Due to this variability and diversity, the fine structure of subtelomeres remains undetermined (Table 1).

The sequence variability of subtelomeres has suggested various possible functions roles of these regions in the stability of chromosomes and their dynamics. Rice subtelomeres, for instance, were involved in transposon movement and recombination (Fan et al., 2008). As shown in rye and wheat, there seems to be a correlation between recombination and chromosome pairing between homologs (Valenzuela et al., 2013). Subtelomeres are frequently affected by recombination events in most systems studied, and these events occur more often in in non-coding regions (Aguilar and Prieto, 2020). The role of subtelomeres in recognition and pairing of homologous chromosomes during meiosis is not well-understood yet. There are some evidences, however, that these regions might be very important, as suggested by a study in Zebra fish, where they found presynaptic subtelomeric chromosome alignment without a fully association of telomeres (Blokhina et al., 2019).

Except for rice and *A. thaliana* (Kuo et al., 2006), the knowledge of the role of subtelomeres in early meiosis is not abundant in plants. Some evidences were found in cereals. Rye subtelomeres showed large clusters of heterochromatic regions at the onset of meiosis (Mikhailova et al., 2001). Wheat has also provided evidences supporting the function of subtelomeres in chromosome pairing. FISH experiments showed that, in the absence of subtelomeric sequences, chromatin remodeling failed and homologous chromosomes would not recognize and pair (Calderón et al., 2014). The relevant function of subtelomeres in recombination was also shown in experiments using wheat lines with distal chromosome deletions (Naranjo, 2015b).

DNA folding and arrangement within the nucleus is also very important for the understanding of the role of subtelomeres in chromosome interactions and pairing. Some of the proteins that are relevant for chromosomes architecture could also be very important, as shown in the case of meiotic cohesins (Zhu and Wang, 2019). Ding et al. (2016) showed that the absence of meiotic cohesins implied a structural change of the chromosome axis, which provoked a failure of homologous chromosomes interaction and pairing. Despite these interesting evidences, further studies are required to demonstrate the actual relevance of the axis formation for homologous chromosome pairing.

A possible role of CTCF, Ying Yang 1, and similar proteins with a known function in chromosome arrangement, has also been suggested in the context of homologous chromosome pairing during meiosis (Beagan et al., 2017). Loop-forming CTCF and cohesins show a similar distribution throughout the chromosome (Wendt et al., 2008). Plant CTCFs, however, have not been identified, though there could be proteins with equivalent functions. Subirana and Messeguer (2011) already mentioned the possibility that HMG proteins could participate in chromosome interaction and homologous pairing.

A recent study of the sequence characteristics of bread wheat distal subtelomeres suggests that the high polymorphism of multiple sequence features, including transposons, retrotransposons, low complexity DNA, and gene-coding sequences, might be responsible for the specificity of interchromosomal interactions at early meiosis, something that is particularly important in a hexaploidy organism like wheat (Aguilar and Prieto, 2020). This study included many other sequence features like the distribution of CpG islands and binding sites of proteins that could play a relevant role in pairing and recombination events. The pattern distribution of all these features seems to be rather specific among heterologous and homoeologous chromosomes (Aguilar and Prieto, 2020).

A study of genes located the in rice subtelomeres revealed a density of 1 gene per 5.9 kbp (Fan et al., 2008). A similar analysis done in wheat showed an average density of 1 gene per 9.5 kbp with a high variability both in density and pattern of gene distribution among the chromosomes studied (Aguilar and Prieto, 2020). Some of these subtelomeric genes that have already been characterized are tRNAs, transcription factor, and metabolic genes. All of them share in common that they represent functions that are required for adaptation to the environment, as suggested by Brown et al. (2010).

The abundance and distribution of transposable elements (TEs) was also chromosome specific in wheat subtelomeres (Aguilar and Prieto, 2020). Several mechanisms could explain the abundance of TEs in subtelomeres, TEs are removed by recombination at a much lower rate in heterochromatic regions, because recombination occurs at a lower frequency in these compacted regions (Zamudio et al., 2015). Besides, TEs density and recombination rate seem to be inversely correlated (Daron et al., 2014). The differential pattern distribution of TEs could support the role of subtelomeres in the specific interactions and pairing of homologs during meiosis.

Plant subtelomeres also include many different repeat sequences, including satellites, simple repeats, and low complexity repeats (Heacock et al., 2004; Torres et al., 2011). In wheat, they account for more than 90% of the entire genome (Li et al., 2004). An analysis of wheat distal subtelomeric region revealed a specific pattern of distribution of these sequences in every chromosome (Aguilar and Prieto, 2020). In maize, tandem repeats are less abundant in the subtelomeric regions but more common across the rest of the chromosome, particularly concentrated in knob regions (Lamb et al., 2007). The abundance and distribution of repeats varies among cereals (Vershinin and Evtushenko, 2014).

In wheat, the overall distribution of repeats, TEs, and genes reveals the same complex and dynamic structure of distal subtelomeres found in all the organisms analyzed, what provides a specificity that could be determinant for homologous chromosome pairing during meiosis (Vershinin and Evtushenko, 2014; Aguilar and Prieto, 2020). However, none of the elements found in subtelomeres is specific of this region, what reinforces the idea that is the pattern of distribution of these elements what is really relevant. Many evidences suggest that subtelomeres play several relevant roles besides chromosome pairing. They could contribute to protect genes located near the chromosome ends and stabilize telomeric regions in the absence of telomeric repeats (Louis and Vershinin, 2005; Garrido-Ramos, 2015).

Other sequence features like GC content and the distribution of CpG islands could also help to understand the role of subtelomeres in chromosome pairing. In bread wheat, these two features showed a great variability among subtelomeres of different chromosomes. GC content is correlated with recombination frequency, which in turn is influenced by homologous chromosome pairing (Sundararajan et al., 2016; Aguilar and Prieto, 2020). A high density of GC content was also correlated with the occurrence of DSBs and crossovers in many organisms (Sundararajan et al., 2016). DSBs seem to be necessary for recombination to take place. The identification of several sequence motifs different organisms suggests that DSBs and crossovers seem to be determined by the presence of specific sequences that could be related to a more relaxed chromatin that facilitates the access of SPO11 and the production of many DSBs (Choi et al., 2018). The analysis of wheat distal subtelomeric regions for the presence of DSB hotspot motifs revealed a good correlation between these sequence motifs and hot recombination spots, with clear differences among chromosomes. A good correlation was also found between density of DSB hot spots and TEs. The differences of sequence patterns among homoeologs subtelomeres in bread wheat point to the possibility that the determinants of chromosome pairing and recombination are related to the very sequence of subtelomeric DNA (Darrier et al., 2017; Aguilar and Prieto, 2020).

Pairing of homologous chromosomes might require the contribution of proteins during the initial stages when chromosomes approach and initiate their interaction (Ding et al., 2016). An analysis of wheat subtelomeres revealed the presence of putative binding sites of some of the proteins that are considered as candidates to play a relevant role in these initial stages of chromosome pairing. Wheat homologous to human SMC1β meiosis-specific cohesin, Ying Yang 1, and HMG were studied (Aguilar and Prieto, 2020). HMG proteins are particularly interesting, since they were suggested to be involved in initial interactions between homologous chromosomes through AT-rich sites (Subirana and Messeguer, 2011). The distribution of putative binding sites for all these proteins showed great differences among wheat chromosomes. An interesting differential pattern of HMG binding sites was revealed, what supports a possible role of HMG proteins during the initial interactions prior to homologous pairing (Aguilar and Prieto, 2020).

## Chromosome Interactions During Premeiosis and Early Meiosis

The spatial distribution of the genome within the three-dimensional nucleus is dynamic during meiosis and linked to regulation of gene expression. Chromosome movements and chromatin remodeling let homologous chromosomes find and associate each other in pairs (Scherthan, 2001; Prieto et al., 2004c; Naranjo, 2014). In most organisms and mainly in plants, chromosomes associate by centromeres at the onset of meiosis, but homologs physically begin interacting through the terminal regions of the chromosomes when the bouquet is formed (leptotene) while all telomeres remained attached to the nuclear envelope. Consequently, the benefit of this telomere cluster on the subtelomeric regions is clear. Subtelomeres have to occupy a very limited space within the nucleus which facilitates the interaction and progressive stabilization of unstable chromosome interactions.

During the process of meiosis, chromosomes need to reorganize and enormously condense, which is crucial for their correct pairing, recombination, and segregation. In mammalian cells, mitotic and meiotic chromosomes show a similar higher-order structure (Kleckner et al., 2013). The higher-order structure of chromosomes is critical in many species (including plants) for diverse cellular processes such as chromosome interactions during meiosis. In meiotic prophase, after DNA replication, chromosomes that are dispersed through the nucleus undergo substantial structural remodeling. When meiosis begins, chromosomes individualize and compact progressively, but pairing, synapsis, and recombination also occurred with their homologous partners. The organization of early meiotic chromosomes is connected with the progression of these interchromosomal interactions, indicating that chromosome morphology is essential for the events mentioned before (Yamada and Ohta, 2013). Chromatin remodeling at the beginning of meiosis is particularly decisive in plants because plant genomes are usually large and complex, carrying a huge number of repetitive DNA, which could allow non-homologous chromosome interactions resulting in chromosome miss-segregation. Chromosome dynamics has been recorded in live maize meiocytes inside intact anthers at the beginning of meiosis showing that chromosomes exhibited an extremely complex dynamic in zygotene and pachytene (Sheehan and Pawlowski, 2009). The observation of different types of chromosome movements at different stages of meiosis in maize meiocytes suggested the existence of multiple mechanisms affecting chromosome mobility, including telomeres attached to the nuclear envelope causing chromosome end movements. Chromosome movements during zygotene in maize illustrate a nice picture on how homologous loci could approach each other in complex genomes allowing chromosomes to search each other based on recombination-dependent homology. Consequently, the dynamic chromosome movements could permit different pairing combinations until correct homologous interactions are successful (Golubovskaya et al., 2002; Sheehan and Pawlowski, 2009).

Two conserved features of meiotic chromosome dynamics, telomeres attached to the nuclear membrane and the random telomeres motion, have been suggested to enable homologous pairing, although their specific functions in meiosis continue to be elucidated. The fact of telomeres being attached to the nuclear envelope might reduce the speed of pairing in contrast with the rates of non-attached chromosomes. Nevertheless, the arbitrarily directed vigorous forces applied to telomeres accelerate chromosome pairing enormously, based on the statistical properties of the telomere force oscillations (Marshall and Fung, 2016). The linker of nucleoskeleton and cytoskeleton (LINC) complexes are important during meiosis. Proteins AtSUN1 and AtSUN2,which are included in the LINC and situated in the internal part of the nuclear envelope, interact with the KASH protein located in the outer nuclear envelope and are implicated in tethering telomeres to the nuclear envelope. As stated before, this attachment contributes to chromosome movements as demonstrated in the double mutant *Atsun1* and *Atsun2* of *A. thaliana*, which showed a delay in prophase I meiotic progression, incomplete synapsis and deficiencies in recombination that result in unbalanced gametes and sterility (Varas et al., 2015). Recently, a partial redundant role of OsSUN1 and OsSUN2 in early meiosis has been also reported in rice (Zhang et al., 2020). *Ossun1* and *Ossun2* double mutants revealed drastic aberrations in telomere clustering, homologous pairing, and crossover formation. In rice, OsSUN2 seems to play a more critical role than OsSUN1 in meiosis, being essential for the telomere bouquet formation (Zhang et al., 2020). ZYGO1 also plays a role in bouquet formation during early meiosis in rice (Zhang et al., 2017). So far, the SUN/KASH protein complex that attach telomeres to the nuclear envelope have not been discovered in wheat yet, but the presence of the *Ph1* locus affects the dynamics of telomere bouquet formation by delaying it, what might imply that chromosomes have more time to check potential pairing facilitating correct homologous chromosome pairing (Richards et al., 2012).

The increased rate of initial pairing at the distal chromosome regions does not only depend on chromosome elongation but instead seems to be also connected with irregular distribution of subtelomeric regions. Hence, active motion of telomeres drives optimal pairing in subtelomeric regions. The distribution is more irregular at the subtelomeres than at the telomeres themselves, according to the results showing that initial pairing rates are highest in subtelomeric sites (Marshall and Fung, 2016). These observations mean that cytoskeletal forces applied on telomeres can regulate abnormal diffusion of subtelomeric chromatin to increase the rate of collisions. Both the limitation of the irregular diffusion to subtelomeres and the initial pairing occurring most likely in subtelomeric regions, when telomeres undergo insistent random walks can describe why in some species, specific “pairing centers” that mediate homologous pairing tend to be located toward the chromosome ends (Marshall and Fung, 2016).

The molecular mechanism explaining how homologous chromosomes associate in pairs at the onset of meiosis as a prelude to recombination remains poorly understood although accurate homologous chromosome associations at the beginning of meiosis are prerequisite for successful recombination between homologs and ensure plant fertility. Chromosome remodeling in meiosis initiates in leptotene stage, when the DNA condenses and sister chromatids are firmly attached (Remeseiro and Losada, 2013). During leptotene, chromatin fibers are looped and anchored to axial elements at the core of the chromosomes (McNicoll et al., 2013). Recombination also begins at this stage. Thus, SPO11 produces DSBs into DNA (Keeney, 2008) and the ends contiguous to these breaks are bound by RAD51 and DMC1 (San Filippo et al., 2008). This process is supposed to be an important feature in homologous recognition in most species. Moreover, the pattern of meiotic recombination has been interpreted as evidence of premeiotic pairing.

Premeiotic homologous pairing has been described in higher eukaryotes such as *Drosophila melanogaster*, suggesting implications for DSB repair in premeiotic cells (Rong and Golic, 2003). In plants, premeiotic homologous pairing has been described in the cultivated rice *O. sativa* and a wild relative *Oryza punctata* (Prieto et al., 2004b). Multiple evidences suggest that chromosome pairing and crossing over are not totally codependent (Jordan, 2006; Zickler and Kleckner, 2015; Calderón et al., 2018). There must be a characteristic of the genomic architecture that could facilitate the processes of recognition and pairing between homologous chromosomes independently of recombination and DNA damage. HMG proteins could participate in these processes by interacting with AT-rich sites, which might be accessible in the expanded DNA loops (Subirana and Messeguer, 2011) and should be studied in the subtelomeric regions in detail. This theory could fit with a mechanism to stabilize the associations between homologs through pairing proteins that interact with AT-rich DNA regions accessible within the DNA protruding loops. Nevertheless, the initial interactions between homologs at the chromosome ends to recognize each other to pair and the molecular factors involved are still unclear, although several genes like HOP1, REC8, and RED1 have been suggested playing essential functions in chromosome associations (Coutou et al., 2004; Jordan, 2006; Ding et al., 2016). A recent mathematical model in polyploids supporting this hypothesis suggested that telomeres are engaged under active forces in a tug-of-war against zippering (Marshall and Fung, 2019). Thus, homologous chromosome regions are competing for zippering with homoeologous regions when telomeres are attached to the nuclear envelope and shaking. Zippering of true homologs is only allowed when the affinity between the distal chromosome regions is strong enough to oppose shaking. This hypothesis agrees with the observations that sequence specificity is essential for the pairing process, essentially in chromosome regions like subtelomeres where DNA sequences are exposed to rapid change (Calderón et al., 2014).

**Figure 1:**
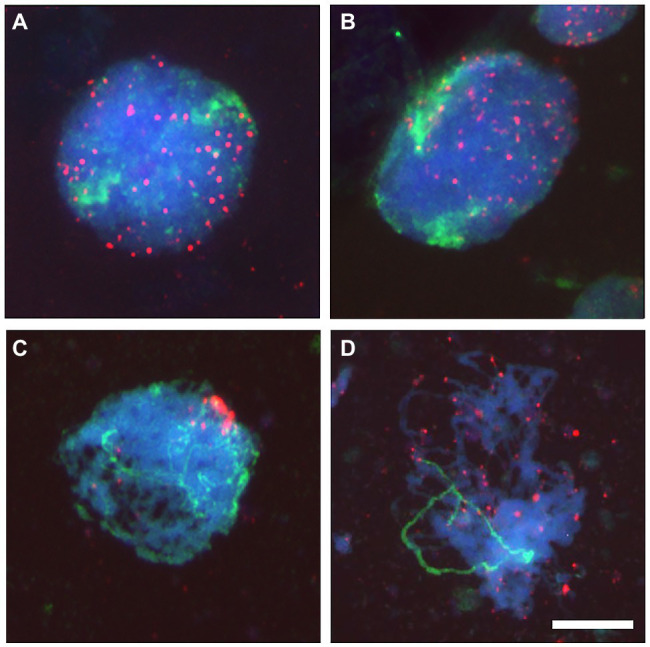
Telomeres dynamic at the onset of meiosis in a wheat line carrying a pair of homologous chromosomes from the wild barley *Hordeum chilense*. Telomeres (red) and *H. chilense* chromosomes (green) were detected in fluorescence *in situ* hybridization (FISH) experiments in wheat meiocytes. Total genomic DNA was counterstained with DAPI (blue). **(A)** Early meiotic nucleus with all telomeres dispersed. Barley chromosomes are occupying different regions within the nucleus. **(B)** As meiosis progresses, telomeres start associating and physically located in one side of the nucleus. **(C)** Early meiotic nucleus with the telomeres clustered in a bouquet. Homologous barley chromosomes are intimately interacting and associating in pairs from the telomeric region. **(D)** Telomeres disperse from the bouquet and homologous chromosomes remained associated in pairs. Bar 10 μm.

When prophase enters early zygotene, DNA fibers expand and chromatin surface becomes more complex (Dawe et al., 1994). Telomeres cluster at the nuclear envelope into the bouquet and heterochromatic knobs elongate (Scherthan, 2007). As stated earlier, the telomere bouquet has been observed in most plants, animals, and fungi, including budding and fission yeast, mouse, wheat, and maize, among others (Martínez-Pérez et al., 2003; Prieto et al., 2004c; Sheehan and Pawlowski, 2009). In wheat, telomeres are spread around the nucleus and at the onset of meiosis start associating in one side of the nucleus, opposite to centromeres, to form the bouquet (Figure 1; Martínez-Pérez et al., 2003). Although many cytogenetic analyses have clearly shown the formation of the bouquet during early meiosis, little evidence about the molecular mechanism to form the telomeres bouquet is available as mentioned before. In addition, though the bouquet itself is not a general characteristic in all organisms, chromosomes associate in most of them by specific regions (telomeres or pairing centers) in a small region of the nucleus (nuclear envelope or nucleolus). Moreover, these telomeres or pairing centers use cytoskeletal elements to perform chromosome movements around this region, and sometimes all telomeres gather within an even smaller bouquet region.

Cell live imaging has been used to visualize chromosome dynamics, but only a few works have been carried out in plants to observe meiosis in real time. The quantification of meiotic phases with high temporal resolution, the diverse chromosome movements during prophase I, as well as some information related to spindle dynamics and chromosome paring have been described in live meiocytes of maize (mentioned earlier) and *Arabidopsis* by visualizing whole chromosomes and microtubules (Sheehan and Pawlowski, 2009; Nannas et al., 2016; Prusicki et al., 2019). Cell live imaging to show telomeres dynamics using a novel CRISP-dCas9 system has been carried out in plant somatic tissues in *Nicothiana benthamiana*. This approach was also combined with fluorescence-labeled proteins and revealed long-range chromatin movements occurring during a short period of time in somatic cells (Dreissig et al., 2017).

In meiosis, how telomeres move along the nuclear envelope and associate in the bouquet, as well as the relative roles of telomeres diffusion and direct movements have been studied using a combination of fluorescence microscopy with mathematical modeling in wheat as described earlier. Sister chromatid telomeres were always found associated to a randomly orientated hemisphere of the meiocytes nuclear envelope and associated in pairs before the telomere bouquet formation (Richards et al., 2012). Such initial telomere associations have also been described in rye (Carlton et al., 2003), maize (Golubovskaya et al., 2002), and rice (Prieto et al., 2004b). The mathematical model mentioned earlier, which incorporates the dynamic of telomere cluster moving along the nuclear envelope, did also include the study of the mechanism of telomeres bouquet formation (Marshall and Fung, 2019). It provides a natural explanation of the pure drift of telomeres to associate and form the bouquet. Although telomeres diffusion might occur, it would be negligible (Richards et al., 2012). In the simplest version of the model, telomere cluster moves with constant drift speed toward the bouquet site (Figure 1). Diffusion is not enough to explicate the deviation in the time of the bouquet formation and directed movements are also required (Carlton et al., 2003; Richards et al., 2012). Thus, a substantial organization of the cytoskeleton (or some other similar structure) is required, creating a grid along which telomeres can move toward the bouquet spot. It is unclear which structural elements are involved in these plant species because SUN/KASH proteins, which link telomeres through the nuclear envelope, have not been described in wheat and in most of plant species. Other possibilities include microtubules (as in animals), although the process in rye does not involve microtubules (Cowan and Cande, 2002), actin (as in *Saccharomyces cerevisiae*), nuclear envelope structural proteins (like the nuclear lamins in animals), or perhaps even the controversial idea of a nuclear matrix.

**Figure 2:**
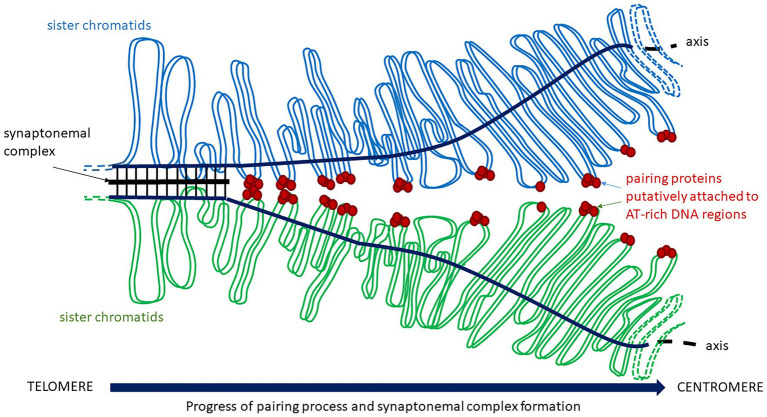
Chromosome pairing and synaptonemal complex formation. Homologs approach and start their interaction putatively through pairing proteins attached to AT-rich regions on DNA loops before the assembly of the synaptonemal complex.

Little information about the molecular mechanisms by which chromosomes specifically recognize a partner to correctly associate in pairs is available, although it has tremendous implications on chromosome dynamics (as described before) and homologous recombination. Recognition between homologous chromosomes must happen at the onset of meiosis and, especially in plant polyploids, it must be highly controlled, because each chromosome has to discriminate its homolog not only from other chromosomes but also from the homoeologous chromosomes of the related genomes. Experiments involving recognition and pairing processes between chromosomes during meiosis are still difficult because these processes are extremely dynamic, occur only among some chromosome regions and are not synchronized from one nucleus to the other (Zickler, 2006). In the context of meiosis, the term “pairing” denotes homologous associations occurring before the formation of the synaptonemal complex, which stabilizes homologs for synapsis and recombination. In fact, the pattern of meiotic recombination has been interpreted as evidence of premeiotic pairing, as it has mentioned before. There must be a feature of the genomic architecture that might facilitate chromosome recognition and pairing independently of recombination and DNA damage (Figure 2). As stated earlier, HMG proteins might participate in homologous chromosome (Subirana and Messeguer, 2011) and should be studied in the subtelomeric regions in detail.

During early meiosis, chromatin decondensation and chromosome movements allow homologs to find each other to associate in pairs (Scherthan, 2001; Prieto et al., 2004c; Naranjo, 2014). In most organisms, and particularly in plants, chromosomes start interacting physically at the bouquet stage, and telomeres being associated to the interior of the nuclear envelope (Figures 1, 2). DNA regions adjacent to telomeres (subtelomeres) might take advantage of this telomere cluster because they are obligated to be in a limited space meanwhile the instigation and progressive stabilization of chromosome interactions occur. The focus on subtelomeres, which are adjacent to telomeres, is an exciting area of study although the polymorphic nature of these regions represents a challenge from a technical perspective. Subtelomeres are less evolutionary conserved than telomeres and include recombination hot spots among other features that complicate the picture of the potential conserved functions of these high-polymorphic regions (Linardopoulou et al., 2005; Louis and Vershinin, 2005; Emden et al., 2019). These DNA segments and their associated proteins are essential for genome stability (Rietchman et al., 2005; Emden et al., 2019).

The implications of subtelomeric regions in chromosome recognition and pairing have been evaluated using wheat lines carrying a pair of homologous chromosomes with terminal deletions from wild barley (Calderón et al., 2014). *In situ* hybridization experiments in these wheat lines clearly showed a function of subtelomeres in the initial processes of homologous recognition and pairing at the onset of meiosis. Although telomeres were present, in the absence of the subtelomeric sequences, chromosome recognition between homologs did not occur and consequently, chromosome pairing is not initiated (Calderón et al., 2014). In addition, in the chromosome arms without subtelomeres chromatin remodeling also failed, though the pairing signal could be conducted from the other chromosome end where subtelomeres were present and successfully initiated chromosome pairing. These observations also contributed to explain the lack of recombination in these terminal regions of the chromosomes (Calderón et al., 2014). According to this, the deficiency in recombination in the terminal region of chromosomes was also confirmed in wheat lines having a deletion at the distal region of any chromosome arm, which did not recover the level of chiasma frequency reached by the intact chromosome (Naranjo, 2015b), supporting the importance of the subtelomeric regions in recombination.

In some species, once homologous chromosomes have associated, the stabilization of both chromosomes depends on the formation of chromosome axis and the synaptonemal complex. In addition, DSB breaks, recombination, and crossover are also needed (Zickler and Kleckner, 2015; Barzel and Kupier, 2018). However, there are numerous indications suggesting that chromosome pairing and crossover are, at least, not completely co-dependent. Pairing can proceed without DSB creation and it can also occur, sometimes between homoeologs, without a subsequent crossover (Zickler and Kleckner, 2015; Barzel and Kupier, 2018; Calderón et al., 2018). This again means that there must be a characteristic on the chromosome architecture that might facilitate recognition, pairing, and recombination without DNA damage. For example, in the absence of homologous chromosomes in the wheat background, homoeologs can pair along their full length although crossing over does not occur in the presence of the *Ph1* locus but in its absence (Calderón et al., 2018; Calderón and Prieto, 2021).

In summary, when a chromosome finds a homolog to associate, their axial elements, now called lateral elements, are linked by the central element of the synaptonemal complex (Fraune et al., 2012). During zygotene, recombination is solved *via* crossovers or non-crossovers (Muyt et al., 2009). Chromosomes continue condensing through diplotene, the synaptonemal complex disappears and the homologs remain together as bivalents through crossovers, which are cytological visualized as chiasmata. It is clear that the importance of terminal chromosome regions including telomeres and subtelomeres, playing crucial roles on chromosome dynamics and interactions during early meiosis in plants.

## Implications of Chromosome Dynamics in Plant Breeding

Exploitation of the whole range of available genetic diversity in plant species could help plant breeders to develop new crop varieties that will be needed in the future to feed the increasing human population. The ability of one chromosome to specifically recognize and associate in pairs only with its homolog, as we have seen before, is a success of meiosis to ensure plant fertility but it is a tremendous barrier that plant breeders need to overcome. Breeders develop inter-specific genetic crosses between the cultivated variety and related species to introduce desirable genes from exotic germplasms into the crop. But in the case of wheat, for instance, sexual hybridization between wheat and related species usually generates interspecific hybrids that contain a haploid set of each parental. This means that wheat and wild relative chromosomes do not usually associate and recombine in many such hybrids. In the context of breeding, it is necessary to shed more light on interspecific associations by the distal chromosome regions and recombination in hybrids or in interspecific genetic crosses, which are developed with the aim of introgressing necessary agronomic characters from related species into crops such as wheat.

Alien chromosome additions have a significant use both in breeding and in plant genetic studies. The specific genetic and cytogenetic properties of DNA introgressions into a crop make these plant materials useful tools for fundamental research, helping to explain the processes of interactions and associations at the distal chromosome regions during specific processes such as meiosis, homoeologous recombination, distribution of specific markers or repetitive DNA sequences, and regulation of gene expression (Chang and de Jong, 2005). For example, in hexaploid, wheat has been developed chromosome introgressions (additions) of both cultivated (*H. vulgare*) and wild (*H. chilense*) barley (Miller et al., 1982; Islam et al., 1978, 1981). These addition lines have a huge potential for plant meiosis studies. For instance, one specific chromosome pair or just a chromosome section can be studied in the wheat background using genomic *in situ* hybridization (GISH) and, consequently rearrangements and interactions can be also analyze uniquely at the distal chromosome regions in a pair of homologous chromosomes (Naranjo et al., 2010; Rey et al., 2015b).

The analysis of the terminal chromosome regions is greatly important in a breeding framework, as telomeres and subtelomeres drive chromosome movements facilitating chromosome interactions, homologous pairing, and consequently recombination. In addition, crossovers are usually located at the terminal region of the chromosomes, as we have described before. In the case of a plant polyploid species such as wheat, pairing and recombination between wheat chromosomes and those from related species carrying desirable traits are suppressed because of its big genome stability, which have adverse effects in a plant breeding framework. Thus, it is crucial to study the effect of terminal regions including telomeres and subtelomeres on chromosome recognition and pairing in the framework of plant breeding. In the polyploid wheat background, addition lines of an extra wild barley pair of homologous chromosomes with terminal deletions are also available (Said et al., 2012). *In situ* hybridization in meiocytes in early meiosis was carried out in these wheat lines to shed light on the subtelomeres effect on the initial processes of homologous recognition and pairing at the onset of meiosis. When subtelomeres are absent, homologous chromosomes are not able to recognize each other and cannot initiate chromosome pairing (Calderón et al., 2014). In addition, chromatin remodeling also fail in the arms without subtelomeres, which implies a delay in pairing, although the pairing signal can be conducted from the other chromosome end which carry subtelomeres and can initiate chromosome pairing. These observations also contribute to explain the lack of recombination in these terminal chromosome regions (Calderón et al., 2014). The absence of recombination in the terminal region of chromosomes was also confirmed in wheat lines with a distal deletion of any chromosome arm. As it was mentioned before, the level of chiasma frequency reached in these deleted chromosomes did not reach the one on the intact chromosomes (Naranjo, 2015b), supporting the importance of the subtelomeric regions in recombination.

Several approaches have been exploited to promote and increase chromosome interactions and recombination between non-homologous chromosomes in a breeding framework. The *Ph1* locus is the main wheat locus suppressing homoeologous recombination between alien and wheat chromosomes, limiting the introgression of desired traits from wheat relatives (Riley and Chapman, 1958; Sears, 1976). Pairing can occur between related chromosomes in lines carrying deletions encompassing the *Ph1* locus, but the chromosomes are heavily rearranged, making recombination between the wheat and related chromosomes difficult but possible. However, interspecific recombination between *Hordeum* species and wheat has been reported at the terminal chromosome regions when the *Ph1* locus was not present (Calderón and Prieto, 2021).

Other approaches have been used from the early fifties with the aim of transferring genes from one species to another. For example, Sears (1956) transferred resistance genes from *Aegilops umbellulata* into wheat. The gametocidal genes are also a tool to transfer chromosomal segments into wheat (Masoudi-Nejad et al., 2002). Unfortunately, these techniques create random breaks and fusion between chromosomes; consequently, most chromosome translocations happen between non-homologs, getting genetic duplications or deficiencies which are not genetically equilibrated. Thus, these random chromosome manipulations are not interesting in plant breeding to be used as genetic tools. It is necessary the development of chromosome manipulation methods that might affect homoeologous chromosome interactions and recombination. Thus, it would be possible to generate more stable genetic introgressions which could be genetically compensated and transmitted to the next generation. A better picture to allow the manipulation of chromosome associations and promote interspecific recombination for plant breeding purposes can be provided by improving our insights into the genetic factors controlling chromosome dynamics and associations at the terminal chromosome regions including telomeres and subtelomeres during meiosis in model plant species such as wheat.

## Author Contributions

MA and PP conceived and wrote this review. All authors contributed to the article and approved the submitted version.

### Conflict of Interest

The authors declare that the research was conducted in the absence of any commercial or financial relationships that could be construed as a potential conflict of interest.
